# Association between combined allergic rhinitis and asthma syndrome in children and nitric oxide: mechanisms, clinical significance, and research progress

**DOI:** 10.3389/fimmu.2026.1787801

**Published:** 2026-08-21

**Authors:** Kai Chen, Haonan Lin

**Affiliations:** 1 Department of Pediatrics, Jiaxing Hospital of Traditional Chinese Medicine, Jiaxing, China; 2 Department of Otolaryngology, Affiliated Hospital of Shaoxing University, Shaoxing, China

**Keywords:** children, combined allergic rhinitis and asthma syndrome, exhaled nitric oxide, nitric oxide, nitrosative stress

## Abstract

Combined Allergic Rhinitis and Asthma Syndrome (CARAS) in children is a chronic airway inflammatory disorder with a high prevalence in childhood. In recent years, a growing body of studies has identified a close association between fractional exhaled nitric oxide (FeNO) and nasal exhaled nitric oxide (nNO) and the pathogenesis of CARAS in children. By synthesizing relevant literature retrieved from the PubMed database (1985-2025), this article provides an in-depth analysis of the roles of the NO/cyclic guanosine monophosphate (cGMP) signaling pathway, nitrosative stress, and organelle injury in the pathogenesis of CARAS in children. Meanwhile, it reviews the potential clinical significance of exhaled nitric oxide as a non-invasive biomarker in supporting the diagnosis and therapeutic monitoring of CARAS in children, analyzes the latest research advances and existing controversies, and outlines future research directions, thereby providing a theoretical basis for the optimized clinical management of this disease.

## 1 Introduction

Allergic rhinitis (AR) and asthma represent the most common chronic airway disorders during childhood. The two conditions are often concurrent, and most children diagnosed with asthma are also afflicted with AR. Given that the nasal cavity and bronchi belong to the respiratory system and constitute the upper-lower airway continuum, the inherent correlation between their anatomical architecture and functional properties offers a key physiological foundation for trans-airway inflammatory extension. Hence, this clinical observation corroborates the well-recognized “one airway, one disease” hypothesis (1–3). Against this backdrop, the diagnostic term combined AR and asthma syndrome (CARAS) was proposed, which specifically denotes the concurrent presence of clinical or subclinical allergic manifestations of the upper respiratory tract (AR) and those of the lower respiratory tract (asthma) (4, 5).

As reported by the World Allergy Organization (WAO), the global prevalence of AR in the pediatric population aged 6 to 12 years is estimated at 10%–30%, with the corresponding prevalence of asthma being 5%–15%. Notably, such rates have been on an annual rise in areas experiencing accelerated urbanization (6, 7). CARAS in children, a comorbid phenotype of the two conditions, presents a key pathological signature dominated by Type 2 Helper T Cell (Th2) -type immune inflammation affecting both upper and lower respiratory tracts. This pathological process is manifested by the infiltration of eosinophils and mast cells, as well as the upregulation of key inflammatory mediators such as Interleukin-4 (IL-4) Interleukin-5 (IL-5), and Interleukin-13 (IL-13) (4, 8, 9).

However, given the rising incidence and complex pathological features of CARAS in children, its clinical management remains challenging. In terms of diagnosis, traditional approaches rely on symptom scoring systems—such as the AR and its Impact on Asthma (ARIA) classification, the Global Initiative for Asthma (GINA) grading—and pulmonary function tests. The former are highly subjective, whereas the latter are difficult to implement in infants and young children. In terms of treatment, antihistamines and inhaled corticosteroids (ICS) are currently the mainstay therapies. Yet long-term ICS use may lead to adverse effects like growth retardation, and existing conventional medications can only alleviate symptoms rather than achieve a cure (10–12).

Since nitric oxide (NO) was confirmed as a vascular endothelial relaxing factor in 1986, its role in airway physiology and pathology has gradually been clarified (13). In the pediatric airway, NO is mainly catalyzed and produced by three types of nitric oxide synthases (NOS): neuronal NOS (nNOS), endothelial NOS (eNOS), and inducible NOS (iNOS) (14–16). Crucially, within the framework of the pediatric “unified airway” CARAS model, recent studies have demonstrated that fractional exhaled nitric oxide (FeNO) levels are significantly abnormal in affected children, closely correlating with airway inflammation severity, treatment response, and acute exacerbation risk (17, 18). More importantly, NO acts not only as a marker but also as a key regulator of airway inflammation in pediatric CARAS: it amplifies Th2-mediated allergic responses via the NO//cyclic guanosine monophosphate (cGMP) signaling pathway and directly contributes to disease progression through nitrosative stress-induced organelle damage (19, 20). Importantly, the association between NO and pediatric CARAS differs distinctly from that in adults, further underscoring the need for focused analysis within the pediatric “unified airway” context. First, children’s airway development remains immature, making exhaled nitric oxide metabolism highly susceptible to age-related influences. Second, the detection techniques for exhaled nitric oxide in infants and young children—such as the tidal breathing method and offline collection method—differ considerably from those applied in adults. Third, during clinical treatment, fluctuations in exhaled nitric oxide levels offer more prominent guiding value for optimizing medication dosages (21–24). Most prior reviews regarding exhaled NO and CARAS primarily synthesize data from mixed adult-pediatric cohorts or only separately discuss FeNO or nNO alone, lacking a systematic integration of the dual indicators (FeNO and nNO) and an in-depth interpretation of NO-mediated pathogenic cascades including the NO/cGMP signaling pathway, nitrosative stress and organelle injury specifically tailored to children. Different from previous literature, this article focuses on the pediatric population, systematically reviewing the integrated mechanisms (NO/cGMP signaling pathway, nitrosative stress, organelle damage), combined clinical application of FeNO and nNO, and latest research progress of NO in CARAS, aiming to provide a new perspective for the precise diagnosis and treatment of this disease.

For clarity, we briefly outline our retrieval strategy. Literature spanning 1985–2025 was searched in PubMed using combined keywords: (children OR pediatric), (CARAS OR combined allergic rhinitis and asthma syndrome), (nitric oxide OR FeNO OR nNO), (pathogenesis OR biomarker), with Boolean AND/OR operators. After deduplication, articles underwent title–abstract screening followed by full-text evaluation. Eligible studies included human trials, animal experiments, systematic reviews and meta-analyses focusing on NO mechanisms or biomarkers in pediatric CARAS. Conference abstracts, case reports, adult-only studies and irrelevant papers were excluded.

## 2 Overview of CARAS in children

### 2.1 Epidemiological characteristics

CARAS in children, a common allergic respiratory comorbidity, is epidemiologically characterized by the significant comorbidity of AR and bronchial asthma (4, 25). Globally, 60%–80% of asthmatic children have comorbid AR (26, 27), with regional data confirming this association: 54.93% of asthmatic children in China have AR (35.01% of AR children have asthma) (28, 29), and 42.5% of AR children (45.1% of multi-sensitized cases) in western Turkey have concurrent asthma (30). Collectively, these findings fully demonstrate the tight coexistence of the two disorders in this clinical syndrome. Childhood is a high-risk period for CARAS: AR typically develops before age 6 (prevalence up to 42% in some cohorts by age 6), asthma onset may extend into adolescence/adulthood, and childhood AR increases lifelong asthma risk (31, 32). Gender distribution shifts with age: males aged 0–10 have 1.65 times the prevalence of females, but this reverses (females higher) in 11–17-year-olds, potentially due to adolescent females’ elevated sex hormones enhancing type 2 immune responses (33). Globally, CARAS-related incidence is rising (AR: 3%–19% globally, 0.8%–14.9% in 6–7-year-olds; asthma: >300 million affected, highest in children), highlighting its public health importance (4, 34, 35). Economically, CARAS in children is costlier than single diseases: in the US, annual direct medical costs average $762 (twice that of single asthma), with 2.3 annual school absences per child (increasing parental work-loss costs); it also raises adult acute asthma risk, with adult annual total costs $1,162 (36).

### 2.2 Pathogenesis

**Figure 1:**
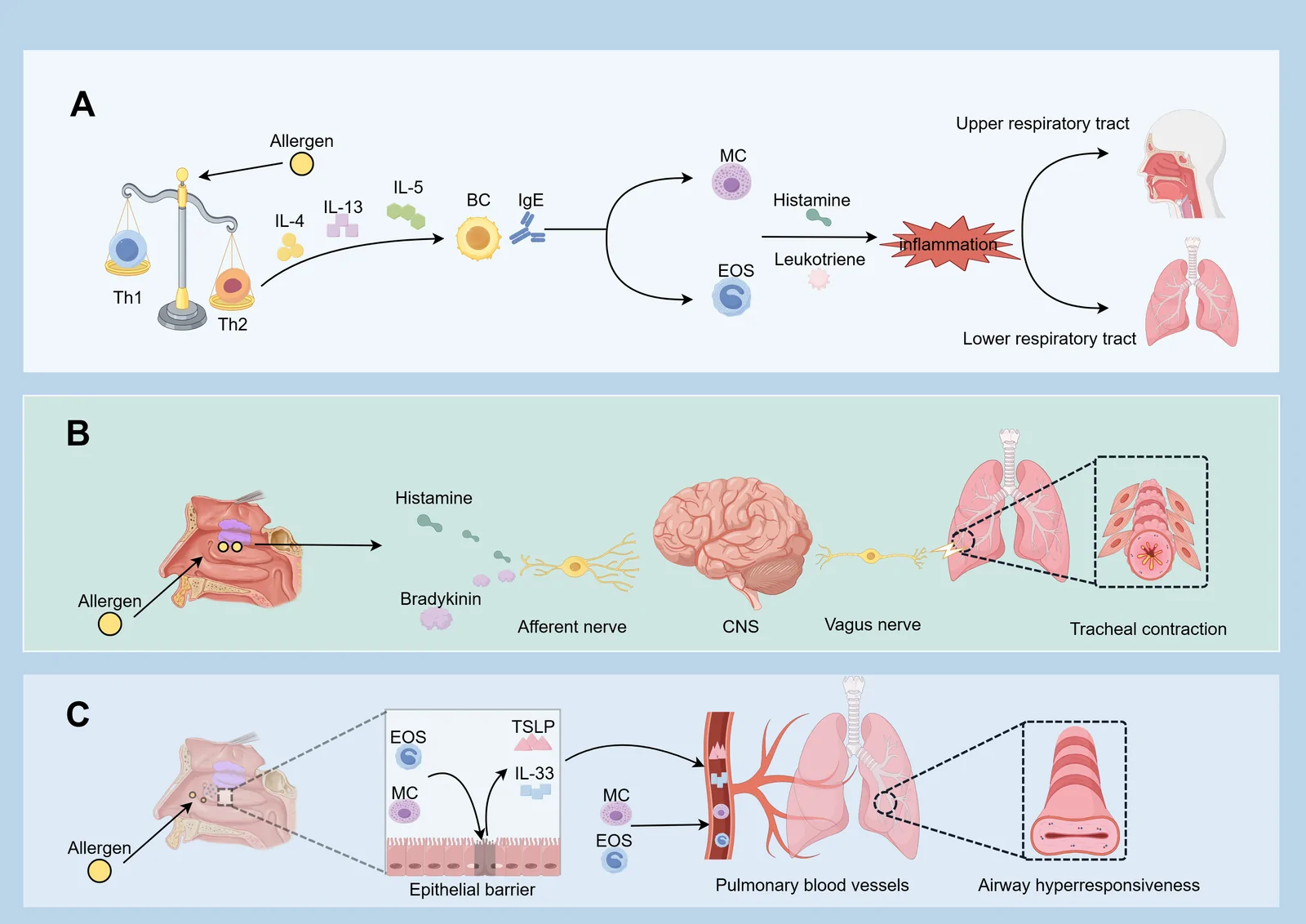
Main pathogenesis of CARAS. **(A)** Allergen exposure disrupts Th1/Th2 immune balance, induces excessive Th2 cell activation and secretion of cytokines (IL-4, IL-5, IL-13), activates mast cells and eosinophils to release inflammatory mediators including amines and leukotrienes, and thereby exacerbates upper and lower respiratory tract inflammation. **(B)** Allergen stimulation of nasal mucosa triggers release of inflammatory mediators (histamine, bradykinin), which activates the afferent nerve-CNS-vagus nerve pathway, induces bronchial smooth muscle contraction and airway narrowing, and ultimately leads to asthma exacerbation. **(C)** Allergen stimulation of nasal mucosa induces activation of inflammatory cells (mast cells, eosinophils) and impairment of mucosal epithelial barrier, which releases alarmins (TSLP, IL-33). These alarmins and inflammatory cells migrate to the lungs via the circulatory system, thereby inducing bronchial hyperresponsiveness. Th1, Type 1 Helper T Cell; Th2, Type 2 Helper T Cell; IL-4, Interleukin-4; IL-5, Interleukin-5; IL-13, Interleukin-13; IL-33, Interleukin-33; BC, B Lymphocyte; IgE, Immunoglobulin E; EOS,Eosinophil; MC, Mast Cell; CNS, Central Nervous System; TSLP, Thymic Stromal Lymphopoietin.

**Figure 2:**
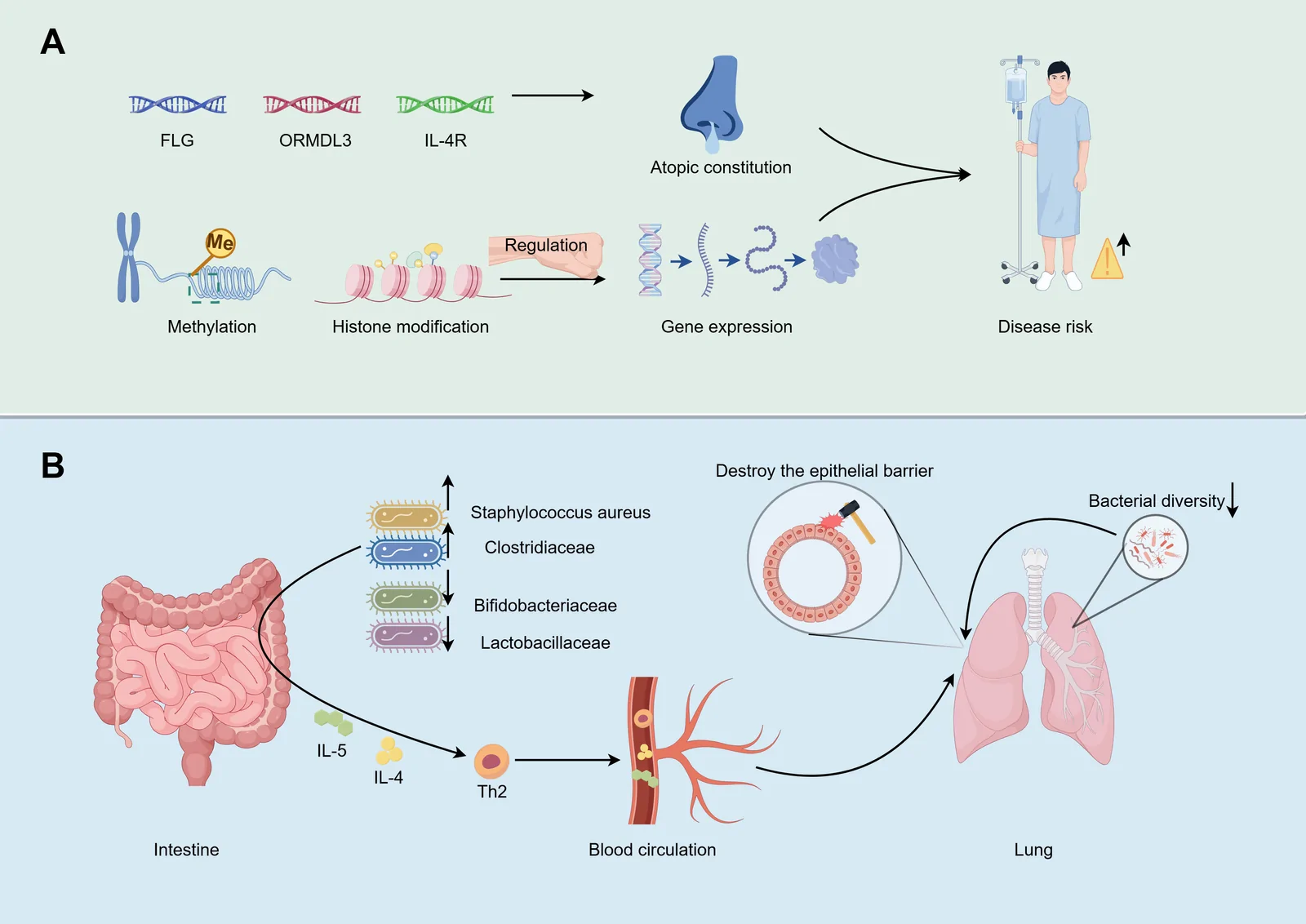
Potential pathogenesis of CARAS. **(A)** Genetic polymorphisms of FLG, ORMDL3, IL-4R et al. predispose individuals to atopic constitution, while epigenetic modifications such as DNA methylation and histone modification upregulate the expression of inflammation-associated genes, thereby increasing disease susceptibility. **(B)** Enrichment of pathogenic bacteria including Clostridiaceae and (S) aureus, accompanied by reduced abundance of beneficial bacteria such as Bifidobacteriaceae and Lactobacillaceae in the gut, induces the release of inflammatory cytokines (IL-4, IL-5) and enhances Th2 immune responses. These inflammatory cytokines and activated Th2 cells migrate to the lungs via the circulatory system, and synergize with local inflammation caused by decreased pulmonary microbiota diversity to jointly impair the integrity of lung epithelial cell barrier. FLG, Filaggrin Gene; ORMDL3, ORMDL sphingolipid biosynthesis regulator 3; IL-4R, Interleukin-4 Receptor; Th2, Type 2 Helper T Cell; IL-4, Interleukin-4; IL-5, Interleukin-5.

The pathogenesis of CARAS in children is complex, with essential mechanisms including Th2-type immune imbalance, the nasal-bronchial reflex pathway, and inflammation migration mediated by epithelial barrier damage. As outlined in **Figure 1**, these are widely regarded as major pathogenic pathways. In terms of immune imbalance, the disorder of Th1/Th2 balance leads to excessive activation of Th2 cells, which secrete cytokines such as IL-4, IL-5, and IL-13, induce B cells to produce Immunoglobulin E(IgE), activate mast cells and eosinophils to degranulate and release inflammatory mediators like histamine and leukotrienes, thereby driving chronic inflammation of the upper and lower airways. Regarding the reflex pathway, allergens stimulate the nasal mucosa to release histamine, bradykinin, etc., which induce bronchial smooth muscle hyperreactivity through the afferent nerve-central nervous system-vagus nerve pathway, triggering lower airway inflammation and asthma attacks. From the perspective of mucosal barrier damage mechanism, allergen stimulation of the nasal mucosa induces the activation and infiltration of inflammatory cells such as mast cells and eosinophils, damaging the nasal mucosal epithelial barrier; the damaged epithelium releases alarmins such as Thymic Stromal Lymphopoietin (TSLP) and Interleukin-33 (IL-33), which, together with immune cells activated in the nasal mucosa, infiltrate the lungs through the blood, aggravating bronchial hyperreactivity and accelerating airway remodeling (4, 37, 38). (**Figure 1**) By contrast, genetic susceptibility, epigenetic modification and microbiota dysbiosis are emerging candidate mechanisms summarized in **Figure 2**, requiring further clinical and experimental validation. Genetic and epigenetic regulation, together with microbial dysbiosis, constitute key potential pathogenic mechanisms. On the genetic side, polymorphisms in genes such as Filaggrin (FLG), ORMDL sphingolipid biosynthesis regulator 3 (ORMDL3) and Interleukin-4 Receptor (IL-4R) are among several known genetic variants that predispose individuals to an atopic diathesis. On the epigenetic side, modifications including DNA methylation and histone modification elevate disease risk by modulating the expression of inflammation-associated genes (38, 39). In the case of microbial dysbiosis, reduced diversity of early-life intestinal or airway microbiota (e.g., decreased levels of Bifidobacterium and Lactobacillus) and enrichment of pathogenic bacteria enhance the propensity for Th2-type inflammation. This disrupts the establishment of immune tolerance and ultimately triggers coordinated allergic inflammation affecting both the upper and lower airways (38, 40) (**Figure 2**).

### 2.3 Current status of clinical management

The current treatment of CARAS in children follows the principle of “coordination of upper and lower airways, and equal emphasis on non-pharmacological and pharmacological treatments”, emphasizing the combination of patient education, environmental control, drug therapy and immunotherapy (4, 41).

The core of non-pharmacological treatment is the control of environmental allergens. By continuously (for at least 3 months) eliminating or reducing exposure to environmental allergens such as dust mites, fungi, and animal dander, the risk of disease recurrence can be effectively reduced and the progression of the disease can be delayed (4, 42).

In drug therapy for AR and asthma, intranasal corticosteroids (e.g., mometasone furoate, fluticasone propionate) serve as first-line pivotal treatments for the upper airway manifestations of these conditions. Adjuvant therapeutic options also include leukotriene receptor antagonists (e.g., montelukast sodium) and intranasal antihistamine preparations (e.g., azelastine), in line with established clinical guidelines for AR and asthma. It should be emphasized that intranasal corticosteroids and intranasal antihistamine preparations require regular and continuous administration for at least 2 weeks to adequately improve upper and lower airway-related symptoms in children with CARAS, based on the guideline-directed management of the individual component conditions (AR and asthma) (11, 43).

To address the “treatment gap” stemming from the limited efficacy and side effects of conventional guideline-directed therapies for AR and asthma, immunotherapeutic approaches provide diversified solutions for managing children with CARAS, and these strategies align with established guidelines for the individual conditions. On the one hand, biological agents allow precise targeting and modulation of immune pathways for moderate-to-severe AR and/or asthma, consistent with their approved indications for these individual conditions. Representative examples include omalizumab (an anti-IgE monoclonal antibody) and dupilumab (an anti-IL-4Rα monoclonal antibody). These agents can significantly alleviate clinical symptoms and improve lung function in eligible patients (38). On the other hand, allergen-specific immunotherapy is a key etiological treatment for IgE-mediated type I hypersensitivity in allergic rhinitis and asthma. The mainstream clinically targeted standardized allergens mainly include two categories: house dust mites (divided into Dermatophagoides pteronyssinus and Dermatophagoides farinae) and grass pollen of the Poaceae family represented by timothy grass. The administration routes are divided into subcutaneous immunotherapy and sublingual immunotherapy (44). Its core immune mechanism is as follows: continuous administration of standardized allergens can up-regulate regulatory T cells and regulatory B cells, prompting the cells to secrete IL-10 and TGF-β to inhibit Th2-type inflammatory responses; meanwhile, it induces blocking IgG4 antibodies to competitively bind to allergens, blocks IgE-mediated degranulation of mast cells and basophils, reduces the release of Th cytokines such as IL-4 and IL-5, and reverses Th1/Th2 imbalance fundamentally (44, 45).

In terms of dosing regimens, most allergen-specific immunotherapy preparations require slow gradient dose escalation to induce immune tolerance: subcutaneous immunotherapy requires in-hospital injection, with a complete course of 3 to 5 years, showing stronger immune induction effects, but necessitating frequent follow-up visits, carrying the risk of systemic allergies such as anaphylactic shock, and is contraindicated in patients with uncontrolled asthma; sublingual drops also require dose titration, whereas only several approved standardized sublingual tablets do not need an escalation phase and can directly adopt a fixed daily maintenance dose (44). Sublingual immunotherapy supports self-administration at home, features better overall safety with very few severe systemic adverse reactions, and demonstrates more prominent treatment compliance in pediatric patients (3, 44, 45).

Despite the relative comprehensiveness of the current treatment system for AR and asthma, the clinical management of pediatric CARAS still faces multiple challenges, as CARAS management is largely predicated on the guideline-directed treatment of its two component conditions (AR and asthma). For one thing, some patients experience recurrent disease episodes due to delayed diagnosis or non-standard implementation of guideline-directed AR/asthma treatment (e.g., unauthorized medication discontinuation, inappropriate dosage adjustment). For another, long-term administration of agents such as intranasal corticosteroids—per their use for AR—may lead to local adverse reactions including nasal dryness and epistaxis (46). Owing to the age characteristics of pediatric patients and their poor treatment compliance, ensuring the continuity of long-term guideline-directed therapy for AR and asthma is challenging (47). In addition, according to GINA, certain patients with severe asthma (a central component of CARAS) exhibit eosinopenia or a neutrophilic inflammatory phenotype; these patients show poor responsiveness to conventional asthma medications, which substantially increases the difficulty of managing the asthma component of CARAS and, in turn, the overall clinical management of CARAS (48).

## 3 Biological characteristics and metabolic laws of NO in children’s bodies

### 3.1 Generation and typing of NO

NO is a colorless, odorless, slightly water-soluble gas that acts as both a toxic gas and a key signaling molecule in organisms. In humans, NO production is predominantly mediated by the enzymatic catalysis of NOS, with three major isoforms—nNOS, iNOS, eNOS—all expressed in the respiratory tract and differing significantly in tissue localization, function, and NO production characteristics (49–53). nNOS and eNOS generate pulsatile, physiological levels of NO to regulate normal bodily functions, while iNOS is inducible under inflammatory stimulation and produces sustained, micromolar levels of NO to mediate inflammation-associated pathological processes (54). Critically, NO exerts concentration-dependent biological effects: low concentrations activate the cGMP/protein kinase G (PKG) signaling pathway for physiological regulation, whereas high concentrations induce nitrosative stress and react with superoxide anions to form reactive nitrogen species (RNS), causing cellular DNA damage and participating in inflammatory pathogenesis (55).

Childhood NO exhibits unique biological characteristics closely linked to the developmental regulation of NOS subtypes and age-dependent dynamic changes in nasal nitric oxide (nNO)—key for CARAS in children pathophysiology. In experimental models, iNOS shows the strongest expression in fetal airway epithelium and vascular smooth muscle, eNOS peaks in adult airway epithelium, and nNOS expression is stable across developmental stages (56); human studies confirm eNOS and vascular endothelial growth factor (VEGF) expression peak in the second trimester of pregnancy, underlying fetal respiratory tract development (57). In children, nNO levels rise with age (6–11 years) and decline thereafter (12–18 years), a trend associated with paranasal sinus maturation and adenoid atrophy—age-related changes that directly impact upper airway NO dynamics in pediatric CARAS (58).

### 3.2 Physiological and pathological functions of NO

NO exerts a central regulatory role in the respiratory system via the NO/cGMP signaling pathway—its key effector pathway in CARAS. NO diffuses freely into cells to activate soluble guanylate cyclase (sGC), which catalyzes guanosine triphosphate (GTP) conversion to the second messenger cGMP and subsequent activation of PKG (59). PKG further inhibits bronchial smooth muscle contraction by regulating potassium and calcium channels, a critical physiological mechanism for maintaining airway tone and modulating asthma and other respiratory diseases (60). Pathologically, excessive NO production—driven by iNOS activation under inflammatory stimulation—is a hallmark of respiratory tract inflammation in CARAS. Excess NO potentiates RNS-mediated oxidative damage, induces nitrosative stress, and interferes with calcium channel function, collectively exacerbating airway tissue pathological injury and inflammatory responses (20, 61).

## 4 The association mechanism between CARAS and NO

### 4.1 NO/cGMP signaling pathway: amplifying Th2-type inflammatory response

The NO/cGMP/PKG signaling axis, as a key regulatory pathway in the core immunopathology (Th2-type inflammation) of childhood CARAS, its mediated mechanism of inflammatory amplification has been clearly elucidated: After the activation of T cell receptor (TCR), it initiates the intracellular production of cGMP and PKG signal transduction; on the one hand, PKG mediates calcium influx by regulating dihydropyridine-sensitive calcium channels, precisely regulating the secretion of Th2 cytokines, and driving the initiation and amplification of airway Th2-type inflammation; on the other hand, activated PKG can further regulate the dihydropyridine receptor (DHPR) on the cell membrane, triggering DHPR-dependent calcium ion influx. After the increase of intracellular calcium ion concentration, it activates calcineurin, promotes the dephosphorylation of the transcription factor nuclear factor of activated T cells and its translocation into the nucleus, binds to the gene promoter regions of Th2 characteristic cytokines such as IL-4 and IL-5, and finally drives the transcription and expression of related cytokines, exacerbating chronic inflammatory responses in the upper and lower airways (19). Beyond directly regulating the production of Th2 cytokines, NO also acts as a key molecule in maintaining the Th1/Th2 immune balance. Studies have confirmed that NO can interfere with immune tolerance mediated by regulatory T cells (Treg), thereby influencing the differentiation trajectory and effector functions of helper T cells (62). Notably, this regulatory effect is concentration-dependent: low NO concentrations tend to trigger type 1 inflammatory responses, whereas high concentrations markedly suppress type 1 inflammatory responses; simultaneously, they activate Th2 cell-specific transcription factors and upregulate IL-4-mediated Th2 cell differentiation (62–65).

### 4.2 Nitrosative stress: peroxynitrite-mediated organelle damage

Nitrosative stress refers to the body’s high responsiveness to RNS. This process is characterized by the excessive production of peroxynitrite (ONOO^-^), which leads to the oxidation of proteins, lipids, and DNA, ultimately triggering cell death (20, 61). As a core effector molecule, ONOO^-^ can target multiple organelles including mitochondria, endoplasmic reticulum and Golgi apparatus (20), and interfere with normal cellular physiological functions through multiple pathways to ultimately induce cell death: it inhibits the activity of key enzymes in the mitochondrial respiratory chain, impairs cellular energy metabolism and triggers mitochondrial apoptosis; its accumulation in the endoplasmic reticulum disturbs protein folding and trafficking, thereby eliciting endoplasmic reticulum stress; it mediates oxidative damage to Golgi proteins, leading to structural and functional disorders of the Golgi apparatus (20, 66–68).

Although no direct evidence has confirmed that ONOO^-^ acts directly on the mitochondria of AR cells, elevated mitochondrial oxidative stress and reactive oxygen species (ROS) levels can markedly promote the generation of RNS, thereby inducing mitochondrial damage and a cascade of subsequent complications (69). Separately, other studies have demonstrated that inhibiting the NOD-like receptor protein 3 inflammasome can enhance mitophagy in nasal epithelial cells and alleviate inflammation in AR mice (70).

In the pathogenesis of asthma, excessive accumulation of ONOO^-^ represents a pivotal factor that triggers respiratory epithelial injury and drives disease progression. When respiratory epithelial cells from asthmatic patients are exposed to excessive ONOO^-^, this anion not only directly induces cellular apoptosis and necrosis, but also activates mitogen-activated protein kinase -mediated signal transduction cascades, thereby further amplifying the epithelial damage effect (71). This damaging process has also been validated in animal experiments: studies have confirmed that ONOO^-^ can induce injury to respiratory epithelial cells in guinea pigs and ultimately elicit airway hyperresponsiveness, a hallmark pathological feature of asthma (72).

From a mechanistic perspective, the targeted damage to organelles by ONOO^-^ represents a pivotal step in its mediation of asthma pathogenesis: it directly impairs mitochondrial function in bronchial epithelial cells, inhibits cytochrome c oxidase activity, disrupts mitochondrial ultrastructure, and induces DNA strand breaks in epithelial cells simultaneously. Ultimately, such organelle dysfunction leads to cellular injury and exacerbation of inflammatory responses (73). Furthermore, intervention experiments using L-arginine have provided further corroboration for this mechanism. By reducing ONOO^-^ generation, L-arginine can selectively restore mitochondrial ultrastructure and alleviate DNA strand breaks in epithelial cells, thereby effectively ameliorating the pathological manifestations of experimental asthma. This finding conversely verifies the central role of ONOO^-^-mediated organelle damage in the development of asthma (73).

### 4.3 NO-mediated linkage of upper and lower airway inflammation

NO plays a complex regulatory role in the linkage of upper and lower airway inflammation, and its functions and related mechanisms have been supported by multiple studies. There is a hypothesis that NO produced in the upper respiratory tract has a protective effect on the entire respiratory tree and can regulate lower airway reactivity (74, 75). In 1992, Corren et al. found in their research that nasal stimulation with allergens could increase the reactivity of the lower airway in patients with AR and asthma (76). However, there is controversy regarding the association between NO and the nasal-bronchial reflex. Some studies have not found a correlation between the levels of nNO, FeNO, and the decrease in forced expiratory volume in 1 second (FEV1) (77). A study involving 317 children and young asthma patients showed that higher nNO levels were significantly correlated with bronchial hyperreactivity, FeNO, and blood eosinophil count (B-Eos) (78).

NO facilitates the transport of inflammatory mediators, representing a pivotal mechanism that mediates inflammatory crosstalk between the upper and lower airways. Experiments involving influenza virus inoculation in iNOS gene knockout mice have confirmed that locally elevated NO levels in the airways can drive trans-airway propagation of inflammatory responses via epithelial cell-mediated pathways (79, 80). Clinically, it has been observed that exhaled NO levels in patients with AR rise markedly following pollen exposure and decline after pharmacological intervention, indicating that NO acts as a key mediator of upper respiratory tract inflammation (81). This phenomenon may be associated with the secondary effects of nasal allergen stimulation on the lower airways, where such triggers can induce lower airway airflow limitation, enhanced eosinophilic airway infiltration, and even a delayed reduction in peak expiratory flow (76, 82, 83).

Modulating the nasal-bronchial reflex, NO lowers the action potential threshold of trigeminal nerve endings to enhance their sensitivity to mild stimuli such as allergens and amplify the nasal-bronchial reflex (46). The pathogenic role of this reflex is widely recognized; stimulation of nasal afferent nerves has been shown to induce significant bronchoconstriction and associated respiratory responses (84). Additionally, a study involving healthy non-smokers demonstrated that mechanical stimulation of the nasal septum or inferior turbinate led to a reduction in FEV1 among participants (85), further validating the objective existence of the nasal-bronchial reflex and the pathogenic role of relevant stimuli.

## 5 The clinical significance of NO in CARAS

### 5.1 NO detection breaks through the limitations of traditional diagnosis

The traditional diagnostic methods for CARAS in children have many limitations, while NO-related detection provides an important supplement for clinical diagnosis and evaluation, with significant clinical significance. (**Table 1**) Traditional diagnostic methods include allergen testing, nasal or airway allergen provocation tests, and lung function and nasal resistance tests, but their clinical applications have significant limitations: nasal allergen provocation tests and bronchial provocation tests are age-restricted and are usually only applicable to children aged 5 and above (46, 86–89). Pulmonary function tests and blood tests have higher requirements for children’s cooperation (90–93). The correlation between the results of rhinomanometry and children’s subjective feelings of nasal congestion is weak, making it difficult to fully reflect clinical symptoms (46, 94); furthermore, the accuracy of skin prick tests in the infant population is suboptimal (95). Conventional pulmonary function tests are also poorly tolerated in young children, and as a practical alternative, impulse oscillometry (IOS) has been widely used for preschoolers. IOS delivers pressure oscillations to the airways during tidal breathing via an external generator and measures airway resistance and reactance to assess airway mechanics, elasticity, and peripheral airway function. Requiring only quiet breathing with a mouthpiece and no special maneuvers, it achieves high success rates in preschool children (96). Compared with conventional methods, exhaled nitric oxide testing features non-invasiveness, convenience and high reproducibility, which is more in line with the clinical needs of pediatric populations. Among them, FeNO, a specific biomarker for airway eosinophilic inflammation (97), can accurately reflect the level of local airway inflammation and make up for the deficiency that blood tests are difficult to focus on the local airway region; additionally, this assay requires no complex cooperation from children, is suitable for young children, and effectively overcomes the age and cooperation limitations of conventional pulmonary function tests and bronchial provocation tests.

**Table 1:** Advantages and disadvantages of traditional diagnostic methods for CARAS.

Detection method	Advantages	Limitations	References
Blood Tests (Total/sIgE)	High specificity (medication/skin condition-independent);sIgE for targeted allergens	Invasive procedure; Poor pediatric compliance; Inaccurate local airway inflammation reflection	(90, 93)
SPT	Rapid & convenient; Detects rare allergens	Limited use in severe eczema patients; Not recommended for infants and young children in non-professional institutions	(95)
NPT	Accurate allergen-symptom correlation verification; High safety/repeatability (sensitivity 83.7–93.3%, specificity 72.7–100%)	Age ≥ 5 yr, poor compliance & symptom description accuracy in young children;Drug/env. interference-prone, false neg./pos. Results	(46, 86)
BPT	Objective diagnosis of atypical asthma; Efficacy evaluation	Age-restricted (not for < 5 yr); Restricted by disease & medication	(46, 88, 89)
PFT	Evaluates lower airway function	Age compliance-dependent; Req. combined indicators for CARAS diagnosis	(91, 92)
RMM	Objective assessment of nasal ventilation function	Weak correlation with subjective nasal congestion scores; Cannot replace comprehensive clinical evaluation	(4, 46)

CARAS, Combined Allergic Rhinitis and Asthma Syndrome; sIgE, Specific IgE; SPT, Skin Prick Test; NPT, Nasal provocation test; BPT, bronchial provocation test; PFT, Pulmonary Function Test; RMM, Rhinomanometry; yr, year; neg./pos, negative/positive; env, environmental.

### 5.2 Application of NO in diagnosis and differential diagnosis

In the clinical assessment of CARAS in children, the key NO-associated detection indicators are FeNO and nNO. Specifically, FeNO levels are closely correlated with airway eosinophilic infiltration, serving as an objective marker for allergic inflammation and proving particularly valuable for identifying asthma with the T2 inflammatory phenotype (92). A study conducted among children aged 6–12 years with AR and/or asthma showed that nNO levels were significantly higher in patients with both allergic rhinitis and asthma as comorbid conditions (17). Further studies have validated the high diagnostic efficacy of this indicator, with a sensitivity of 72.97% and an exceptionally high specificity of 95.83% observed in pediatric asthma cases (98). Moreover, alterations in FeNO levels precede changes in clinical symptoms and pulmonary function parameters, a distinctive feature that renders it well-suited for the long-term dynamic monitoring and management of disease status in affected children (81).

At present, research in the English literature regarding the independent diagnostic value of nNO for pediatric CARAS remains relatively scarce. Existing evidence shows that nNO levels are comparable between CARAS patients and those with AR alone, yet concentrations in both groups are markedly higher than in healthy controls and patients with asthma alone (99).

For single-disease diagnosis of AR, nNO exhibits extremely high sensitivity and specificity, with multiple studies validating its excellent diagnostic performance via definitive quantitative thresholds: a 2017 study identified optimal nNO cutoff values of 775 ppb in children and 799 ppb in adults with non-asthmatic AR, reporting a pooled sensitivity of 92.7%, a specificity of 91.7% in children, and a specificity as high as 95.0% in adults (100); a 2022 study established a cutoff of 390.0 ppb, yielding a sensitivity of 73% and specificity of 80% (101); a 2021 study focusing on children with severe dust mite-induced AR proposed a higher cutoff of 1350 ppb, with a sensitivity of 78% and specificity of 71% (102).

In clinical practice, FeNO levels decline rapidly with inhaled corticosteroid therapy, making it a reliable biomarker for assessing treatment response, monitoring medication adherence, and guiding individualized therapeutic regimen adjustments (92). Notably, both FeNO and nNO levels are influenced by age, allergic status, recent infection, and medication use, and thus cannot serve as standalone definitive diagnostic criteria for CARAS; instead, they require comprehensive interpretation in conjunction with clinical symptoms, pulmonary function tests, and other relevant indicators (91, 103). Further, combined FeNO and nNO testing enables simultaneous evaluation of upper and lower airway inflammatory status: children with CARAS typically show concurrent elevation of both indices, while those with isolated AR or isolated asthma predominantly present with isolated nNO or FeNO elevation, respectively (99). This combined testing approach helps clarify the scope of airway inflammatory involvement and provides critical reference for the precise diagnosis of pediatric CARAS. To intuitively summarize the diagnostic efficacy of FeNO and nNO in different airway allergic diseases in children, the clear cut-off values, sensitivities, and specificities from existing studies are collated in the following table (**Table 2**).

**Table 2:** Diagnostic efficacy of children’s FeNO and nNO in different airway diseases.

Detection indicators	Study population (children)	Diagnostic cutoff value	Sensitivity	Specificity	References
FeNO	Asthma (T2 inflammatory phenotype)	>20 ppb (general threshold for children)	81% (efficacy monitoring)	80% (efficacy monitoring)	(18, 51, 92)
FeNO	Healthy children (without asthma/atopic diseases)	<25 ppb (upper limit value)	–	–	(51)
nNO	CARAS	–	72.97%	95.83%	(17)
nNO	AR single disease entity	–	83% (base value); maximum 100%	80% (base value); maximum 94.9%	(100)
nNO	CARAS/AR single disease vs healthy control/asthma single disease	No unified threshold (all are significantly higher than the latter two)	–	–	(99)

FeNO, fractional exhaled nitric oxide; nNO, nasal nitric oxide; AR, Allergic rhinitis; CARAS, Combined Allergic Rhinitis and Asthma Syndrome.

“-” indicates that the relevant index has not been reported or determined in the existing studies, and no available data can be provided.

### 5.3 Treatment monitoring and efficacy evaluation

Exhaled nitric oxide-associated parameters can serve as non-invasive biomarkers for efficacy evaluation and dynamic monitoring of treatment responses in CARAS in children. The 2023 International Consensus Statement on AR explicitly states that FeNO levels in children decrease significantly following standardized pharmacotherapy, while nNO levels also exhibit a downward trend after appropriate anti-allergic treatment—findings that fully support the potential utility of both indicators for monitoring responses to anti-inflammatory therapy (46). As a well-established non-invasive marker of airway inflammation in children, the responsiveness of FeNO to anti-inflammatory treatments has been validated and thoroughly reviewed in existing literature. For instance, following treatment with ICS, FeNO levels decline markedly in parallel with the resolution of airway eosinophilic inflammation. This parameter also plays a pivotal role in monitoring disease progression and assessing treatment responses in asthmatic children, particularly those with the eosinophilic phenotype. When the FeNO cutoff value is set at > 17 ppb, its sensitivity and specificity for efficacy monitoring reach 81% and 80%, respectively (18). Furthermore, FeNO possesses notable long-term predictive value: FeNO levels during infancy can effectively predict the risk of persistent asthma and acute exacerbations in school-aged children, indicating its potential as an early-warning biomarker for future asthma episodes (104). A subsequent study focusing on children aged 3–7 years further confirmed that FeNO is predictive of asthma control status, and that uncontrolled AR constitutes a key factor influencing FeNO levels (105).

Mechanistically, airway epithelial cells constitutively express iNOS. Upon stimulation with Th2-type cytokines such as IL-13, iNOS expression is upregulated, which in turn enhances NO production and subsequent exhalation—thus enabling FeNO levels to indirectly reflect the inflammatory status and functional integrity of airway epithelial cells (106). Additionally, an exploratory analysis of a 2022 randomized controlled trial demonstrated that FeNO levels can distinguish distinct clusters of airway epithelial gene expression, further corroborating the close correlation between FeNO and the molecular functions of airway epithelial cells (107). In summary, FeNO and nNO, as key non-invasive biomarkers in childhood CARAS, have had their biological mechanisms, influencing factors, and clinical application value confirmed by numerous studies. To present their core characteristics and application scenarios more clearly, the key information is systematically summarized as follows (**Table 3**).

**Table 3:** Summary table of biological mechanisms, influencing factors, and clinical application value of FeNO and nNO in children with CARAS.

Core biomarkers	FeNO	nNO
Action Mechanism	• Th2 cytokines (e.g., IL-13) upregulate iNOS expression in lower airway epithelial cells, inducing NO synthesis and exhalation (106) • Concentrations positively correlate with airway eosinophil infiltration; indirectly reflect airway epithelial inflammatory status and integrity (92, 106)	• Primarily reflects inflammatory status of nasal cavity and paranasal sinuses, closely linked to sinus mucosal iNOS activity (54, 100, 114, 119) • Participates in upper-lower airway cross-linkage (e.g., nasal-bronchial reflex regulation, inflammatory mediator transport) (46, 78– 80)
Influencing factors	Elevating factors (increase FeNO level) • Allergic disease history (92, 99) • Intake of nitrate-containing food (115–117) Reducing factors (decrease FeNO level) • Tobacco smoke exposure (112, 113) • Older age (12–18 years) (58) • Male sex (103) • Inhaled/systemic glucocorticoid treatment (18, 46, 92)	Elevating factors (increase nNO level) • Allergic rhinitis (92, 99) • Intake of nitrate-containing food (115–117) Reducing factors (decrease nNO level) • Tobacco smoke exposure (blocks sinus ostium, deactivates NO, damages mucosa) (114) • Nasal congestion, sinusitis and other nasal lesions (103, 118, 119) • Age over 12 years (sinus maturation + adenoid atrophy) (58, 108)
Value in clinical diagnosis and monitoring	1. Diagnosis & differential diagnosis (80, 92, 97, 99) • Identifies T2-high asthma phenotype; aids diagnosis of pediatric CARAS (accompanied by elevated nNO) 2. Therapeutic monitoring & efficacy evaluation (18, 46, 92, 106) • Rapidly responds to ICS and other anti-inflammatory therapies; concentration reduction parallels resolution of eosinophilic inflammation • Guides individualized medication adjustment, monitors adherence 3. Prognostic prediction (80, 104, 105, 109) • Infant FeNO levels predict school-age persistent asthma and exacerbation risk • Changes precede clinical symptoms and pulmonary function abnormalities, suitable for long-term surveillance	1. Diagnosis & differential diagnosis (17, 98–100) • High diagnostic performance for allergic rhinitis (sensitivity: 83%–100%) • nNO significantly higher in CARAS children than healthy subjects or isolated asthma patients; comparable to isolated AR, facilitating evaluation of upper airway inflammatory range 2. Therapeutic monitoring (47, 81) • Levels decline after standardized anti-allergy therapy, enabling upper airway inflammation monitoring 3. Combined biomarker assessment (78, 92, 99) • Concurrent FeNO + nNO testing enables simultaneous evaluation of upper and lower airway inflammation, supporting accurate CARAS diagnosis

FeNO, fractional exhaled nitric oxide; nNO, nasal nitric oxide; AR,Allergic rhinitis; CARAS, Combined Allergic Rhinitis and Asthma Syndrome; IL-13, Interleukin-13; ICS, inhaled corticosteroids.

## 6 Research progress and existing controversies

### 6.1 Progress in clinical application

Age-specific exhaled nitric oxid Reference Value Establishment: Developing age-specific exhaled nitric oxid reference values is a key research direction for the clinical assessment of pediatric airway diseases (51). The 2011 American Thoracic Society clinical guidelines propose FeNO thresholds of 20 ppb for children and 25 ppb for adults, yet no standardized FeNO reference range has been established for healthy children to date, and the upper FeNO limit is approximately 25 ppb in healthy children free of asthma and atopic disorders (51).

Similarly, no consensus has been reached on the threshold range of nNO. A study on healthy children showed that the normal nNO level in children aged 6–17 years is (449 ± 115) ppb, and the nNO threshold in children aged 6–12 years is positively correlated with age—each one-year decrease in age leads to an 11.5 ppb drop in the threshold (108). Currently, the direct link between nNO thresholds and specific diseases remains unconfirmed, but existing research has offered important references for its clinical translation: nNO levels are commonly elevated in children with AR (100). In contrast, despite initial progress in developing age-specific nNO reference intervals, there is still much room for research in refined stratification and standardization. Existing studies have clarified the FeNO diagnostic threshold differences between children and adults, defined the FeNO upper limit in healthy children and the normal nNO range in 6–17-year-olds, providing key evidence for clinical evaluation of pediatric airway diseases. However, these indicator measurements are easily confounded by various factors, and diagnostic sensitivity and specificity differ greatly across studies. Thus, it is urgent to establish standardized testing procedures and develop personalized clinical application plans.

The development of portable exhaled nitric oxide detection technology has provided key technical support for long-term home management of pediatric airway diseases. A study of 151 asthmatic children with daily FeNO monitoring showed that continuous home monitoring not only improves children’s asthma control, but also effectively reduces steroid dosages (109), which highlights the application value of portable home NO monitors in long-term childhood asthma management. Currently, mainstream clinical portable NO detectors include the Aerocrine Niox series (including Niox Mino), Medisoft FeNO+, CLD 88 Analyzer, and Vivatmo pro (110). The relative strengths and weaknesses of these four devices are compared in the table below (**Table 4**) Starting from the pivotal needs of home scenarios, such as easy operation, low maintenance costs, and adaptation to daily monitoring, Vivatmo pro is the most suitable for home application among the four devices. This device has a high degree of automation, and also has the advantages of no calibration and no consumables. Moreover, its non-invasive design is highly suitable for home monitoring scenarios, which can effectively provide convenience for long-term family monitoring (110).

**Table 4:** Comparison of core parameters of mainstream clinical portable NO detectors.

Device model	Detection principle	Detection duration	Measurement range ( ppb)	Age group	Clinical validation	Core advantages	Limitations	Adaptive scenarios	References
Aerocrine Niox Mino	Electrochemical method	10–15 s/test	FeNO:5–300; nNO:5–1,700 (There are also data stating that FeNO: 1–300; nNO: 1–1,700 )	≥4 yr, adults	Complies with ATS/ERS; validated in multi-center clinical studies (pediatric cohorts included); repeatability (CV ≤ 13%)	Simple operational steps, compact and portable form facto; built-in NO filter (anti-interference); stable airflow (50 mL/s); minimized dead volume.	FeNO-only, no nNO mode; sensor signal drift, req. regular calibration	Outpatient rapid screening; pediatric asthma long-term home monitoring	(110, 128, 129)
Medisoft FeNO +	Chemiluminescence method	15–20 s/test	0–600	≥5 yr, adults	Fully complies with ERS/ATS; supports multi-center data comparison; European clinical certified	Multi-mode detection (bronchi 50 mL/s, nasal cavity 100 mL/s, alveoli); sync output of NO flow & expiratory pressure.	Larger than Niox series, average portability; high consumable cost	General hospital Respiratory/Allergy Dept; simultaneous upper & lower airway inflammation assessment	(110, 130–132)
CLD 88 Analyzer	chemiluminescence method	Real-time detection (results instant)	0.1–5,000	All ages (infants to adults)	EU clinically approved; fully compatible with ATS/ERS; supports infant tidal breathing detection verification	Provides a minimum detection limit of ≤ 1 ppb; no consumables; continuous NO flow/volume monitoring; lung function comprehensive evaluation software.	Desktop: large size; portable: high cost; professional training req. for operation	Pediatric specialist hospitals & research institutions; infant/young child/special population testing	(110, 129)
Vivatmo pro	Electrochemical method	8–12 s/test	5–300	≥6 yr, adults(including home self-testing)	Demonstrated consistency with standard equipment in clinical validation studies); HL7/GDT medical IT system certified.	Wireless inductive charging; no calibration/consumables; touchscreen operation; medical system data sync; low maintenance cost.	High FeNO (>50 ppb): slight deviation vs. chemiluminescence method; no infant detection support	Long-term family monitoring; patient self-management; community medical follow-up	(110, 129)

yr, year; ATS, American Thoracic Society; ERS, European Respiratory Society; HL7, Health Level 7; GDT, Device Data Transfer; nNO, nasal nitric oxide; FeNO, fractional exhaled nitric oxide; CV, coefficient of variation; EU, European Union.

## 7 Challenges, controversies, and future perspectives

While FeNO and nNO offer partial auxiliary insights for clinical assessment and follow-up of children with CARAS, their utility is constrained by abundant unresolved inconsistencies and practical barriers in clinical application and mechanistic research. These drawbacks greatly restrict their reliable widespread use in daily clinical practice and underline the necessity of further high-quality research to fill current evidence gaps. Multiple controversies, practical limitations, and unmet needs still exist in their clinical translation and mechanistic interpretation, which collectively hinder their optimal application in routine practice and highlight the need for targeted future research efforts.

Key controversies persist regarding the interpretation and mechanistic role of NO-related indices, starting with the widespread interference of confounding factors on FeNO and nNO levels. Both indicators are influenced by AR history, smoking exposure, and dietary nitrate intake: AR may elevate both FeNO and nNO levels (111); smoking exposure reduces FeNO by upregulating arginase and competing with L-arginine metabolism (112, 113), while it decreases nNO via sinus ostial obstruction, chemical inactivation of NO by ROS, and damage to nasal epithelial cells (114); and dietary nitrates contribute to NO production through the nitrate-nitrite-NO pathway, thus affecting both FeNO and nNO (115–117). Additionally, distinct specific influencing factors exist between the two indices—FeNO is specifically affected by age, sex, and glucocorticoid use, whereas nNO is more strongly influenced by nasal conditions such as congestion and sinusitis (103, 118, 119). Notably, there remains no unified correction standard for these confounding variables across existing studies; inconsistent adjustment strategies across cohorts create incomparable cut-off values and generate conflicting diagnostic conclusions, which severely impede cross-study data pooling and unified clinical judgment. Compounding these challenges is the unresolved causal relationship between NO and disease pathogenesis: NO may act as both a downstream marker of inflammation and a potential pathogenic mediator. On one hand, FeNO and nNO increase after allergen challenge and decrease with steroid therapy, reflecting Th2-driven iNOS upregulation ( (92, 120) on the other hand, iNOS gene polymorphisms are associated with the risk of childhood); allergic asthma and AR, suggesting that NO may contribute to disease onset (121–123), and its dual role in allergic inflammation has not been fully elucidated. This dual identity creates contradictory interpretations in current literature: some studies frame NO solely as a passive inflammatory readout, while others emphasize its active pro-inflammatory pathogenic function, and no consensus has been reached to reconcile these two competing hypotheses.

Beyond these controversies, several critical clinical limitations restrict the routine application of FeNO and nNO in children with CARAS. Notably, FeNO has limited value in non-eosinophilic asthma (NEA), which accounts for 30%–40% of pediatric asthma cases; FeNO levels are often below the diagnostic threshold (20 ppb) in NEA, making it insufficient to guide treatment (51, 124). While integrated strategies combining FeNO with blood eosinophils, polyp history, lung function, and simplified classification algorithms have been proposed to address this gap, clinical validation remains lacking (125). Standardized reference values also remain unavailable, particularly for infants and preschoolers—although low-flow measurement and offline tidal breathing methods have improved feasibility in young children (126, 127), age-specific normative data for children under 3 years old have not been established, and heterogeneous measurement protocols further limit the generalizability of existing small-sample studies. Divergent threshold values reported by different pediatric cohorts create conflicting diagnostic recommendations, and no global consensus cut-offs for combined FeNO plus nNO testing in CARAS have been validated to date. Clinical implementation is further hindered by high costs, uneven global accessibility of detection devices, and insufficient standardized clinical protocols; many primary care settings, where the majority of pediatric CARAS cases are managed, lack the necessary equipment and trained personnel, and current guidelines provide limited guidance on adjusting inhaled corticosteroid therapy or determining optimal monitoring frequency based on serial NO measurements. Most importantly, neither FeNO nor nNO can serve as definitive standalone diagnostic biomarkers; they only deliver complementary auxiliary evidence and must be interpreted alongside clinical symptoms, lung function and allergen test results to avoid misdiagnosis or missed diagnosis. Even when applied in combined detection, these two biomarkers cannot independently confirm or exclude CARAS, and their predictive performance varies substantially across pediatric populations.

To address these controversies and limitations and promote the individualized management of pediatric CARAS, future research should focus on high-quality evidence generation and seamless clinical translation. Priority should be given to standardized detection in young children, with large-sample, multi-center studies needed to validate low-flow and tidal breathing methods and establish age-specific FeNO reference intervals for infants and toddlers. For precision phenotyping and treatment guidance, machine learning approaches integrated with multi-dimensional indicators—including FeNO, nNO, B-Eos, allergen-specific IgE, and omics data—should be used to define NO-related inflammatory phenotypes, especially in children with comorbidities such as obesity, preterm birth, or rhinitis, with prospective external validation to facilitate the translation of these models into routine clinical practice. In terms of mechanistic understanding and prevention, longitudinal studies are essential to clarify how air pollution, climate change, and indoor allergen exposure dynamically affect children’s NO levels, while genomic, epigenetic, and multi-omics approaches will help delineate causal pathways between environmental exposures, genetic factors, and NO metabolic abnormalities, thereby identifying precision prevention targets. Finally, efforts to improve clinical implementation should focus on developing cost-effective detection devices, advancing home-based monitoring technologies, simplifying clinical pathways, and establishing guideline-based treatment algorithms that incorporate serial NO changes. Only through such targeted efforts can the full potential of NO detection be realized, translating into improved prognosis for children with CARAS. Only after resolving the above-mentioned methodological, equipment and evidence deficiencies can the auxiliary value of NO detection be reasonably exerted; at present, its clinical potential cannot be overestimated without further robust clinical verification, which is vital for improving long-term prognosis for children with CARAS.

## 8 Summary

As a “unified airway” disease, CARAS pathogenesis is closely associated with abnormal NO metabolism. Under physiological conditions, NO maintains airway homeostasis; under pathological conditions, excessive activation of iNOS results in overproduction of NO, which amplifies Th2-type inflammation via the NO/cGMP signaling pathway or induces organelle damage through ONOO^-^, thereby aggravating airway inflammation and impairing the epithelial barrier. Meanwhile, as non-invasive biomarkers, FeNO and nNO show considerable complementary value in supporting disease diagnosis, treatment monitoring, and prognostic assessment, though their capacity to support mature precision medicine workflows for children with CARAS remains constrained by multiple unresolved research gaps.

Although current studies remain controversial in terms of confounding factor adjustment and causal inference, the routine clinical implementation of NO-guided treatment strategies for pediatric CARAS still faces several key challenges: unsatisfactory cost-effectiveness hinders widespread application; accessibility of detection equipment in primary medical institutions is limited; and validated diagnostic and therapeutic protocols based on dynamic NO changes are urgently needed.

With the advancement of standardized detection technologies, the development of NO-targeted drugs, and the application of artificial intelligence, NO-related research may gradually mitigate these existing barriers, rather than fully resolve all current limitations in the short term, and provide tentative novel approaches to further refine strategies for the management of pediatric CARAS, rather than deliver mature, universally applicable precision medicine regimens at present, ultimately helping to potentially improve the long-term prognosis of affected children after adequate high-quality clinical validation fills the current evidence deficits.
